# Associations between exposure to intimate partner violence (IPV) and infant developmental delay: moderating role of women’s empowerment at six weeks postpartum

**DOI:** 10.1186/s12889-026-26528-9

**Published:** 2026-02-09

**Authors:** Anum Nisar, Soim Park, Rakhshanda Liaqat, Ahmed Zaidi, Abid Malik, Najia Atif, Atif Rahman, Pamela J. Surkan

**Affiliations:** 1 Department of Primary Care and Mental Health, Institute of Population Health, University of Liverpool, Waterhouse Building, Block B First Floor 1-5 Dover Street, Liverpool, L69 3GL United Kingdom; 2 Bloomberg School of Public Health, Johns Hopkins University, Baltimore, USA; 3 Human Development Research Foundation, Islamabad, Pakistan; 4 Health Services Academy, Islamabad, Pakistan; 5 Department of Primary Care and Mental Health, Institute of Population Health, University of Liverpool, Waterhouse Building, Block B First Floor 1-5 Dover Street, Liverpool, L69 3GL UK

**Keywords:** Intimate partner violence, Infant developmental delay, Women’s empowerment, Postpartum period, Early child development

## Abstract

**Background:**

Intimate Partner Violence (IPV) is a significant threat to women’s wellbeing and child development, particularly in lower- and middle-income countries (LMICs). Despite its prevalence, research in LMICs on the effects of IPV on child development remains limited. This study investigates the association between IPV and different domains of child development as well as the moderating role of women’s empowerment.

**Methods:**

Participants included 397 women with at least mild anxiety from a randomised trial of a perinatal anxiety intervention at Holy Family Hospital in Rawalpindi, Pakistan. Child development was assessed at six weeks postpartum using the Ages and Stages Questionnaires (ASQ-3), including communication, gross and fine motor skills, problem-solving, and personal-social domains. At six weeks postpartum, women reported on IPV experiences occurring from the beginning of pregnancy to six weeks postpartum, using items from the Pakistan Demographic and Health Survey, assessing physical (7 items) and emotional (3 items) violence from the beginning of pregnancy to six weeks postpartum. Women’s empowerment was measured using a culturally adapted four-item subscale assessing “freedom from family domination,” which reflects women’s control over household influences and decision-making. Data were analysed using multivariable linear regression. Interaction terms were used to examine whether empowerment at six weeks postpartum moderated the association between IPV and child developmental delay at six weeks postpartum.

**Results:**

Our findings show significant, negative associations between IPV scores and all child development scores (adjusted Bs=-0.64 to -1.00, all *p*-values < 0.05), suggesting poor development. Significant interactions were observed for communication, gross motor, and problem-solving domains (all p-values < 0.05), with marginal significance for the personal-social domain (*p* = 0.05). Among empowered women, the associations were not significant, while non-empowered women showed stronger negative impacts (adjusting Bs=-1.31 — -2.13, all *p*-values < 0.05).

**Conclusion:**

Women’s empowerment may mitigate the adverse effects of IPV on child developmental delay. Empowerment initiatives could improve outcomes for children of mothers experiencing IPV.

**Supplementary Information:**

The online version contains supplementary material available at 10.1186/s12889-026-26528-9.

## Introduction

Intimate Partner Violence (IPV), defined as physical, emotional, or sexual abuse between intimate partners, is a pervasive global issue that significantly affects women’s well-being [1, 2]. The consequences of IPV extend beyond mothers and have significant implications for their children’s health and development [3]. Globally, IPV is recognized as a critical determinant of poor child development [4–6]. Research from LMICs has identified a link between IPV and childhood illnesses, such as acute respiratory infections and diarrhoea, which are among the leading causes of child mortality [7]. A study across 29 LMICs found that exposure to IPV significantly increased the likelihood of stunted growth in children aged 0 to 5 years [8]. In Pakistan, emotional violence against mothers has been associated with poor nutritional status in children under the age of five [9].

In Pakistan, nearly one in three ever-married women reports experiencing physical or emotional IPV, with emotional abuse being most preva[10, 11]. Only about half of children aged 3–5 are developmentally on track [12], and women’s decision-making power remains limited within patriarchal family systems [13]. These intersecting challenges of violence, low empowerment, and suboptimal early development make Pakistan a critical setting for examining their combined effects on child outcomes.

Given the high prevalence of IPV in LMICs, ranging from 20% to 46% [11] and women’s primary role in childcare in these contexts, IPV is likely to have an important impact on child health [14, 15]. While evidence that supports the adverse effects of IPV on child health is growing, research in LMICs remains limited, particularly with respect to a wide range of child developmental indicators beyond physical growth.

The relationship between IPV and women’s empowerment is also complex and not well understood. Women’s empowerment refers to the process by which women gain the ability to make strategic life choices in a context where this ability was previously denied to them [16]. It encompasses dimensions such as economic participation, decision-making power within the household, mobility, access to resources, and self-confidence. Empowerment is a multifaceted phenomenon that can vary across cultures and contexts; for instance, a woman in Pakistan may feel empowered in making household decisions but may lack confidence or authority outside the home. In this study, we conceptualized women’s empowerment as “freedom from family domination,” a dimension that reflects women’s autonomy in household decision-making and resistance to coercive control by family members [16, 17]. This construct is particularly relevant in Pakistan’s extended-family systems, where restrictions on women’s movement, finances, and personal choices often stem from within the household rather than the broader community. We prioritized this dimension as it captures everyday expressions of power and control most salient to women’s lived experiences in this context.

Several reviews have demonstrated mixed findings, with some studies showing that empowerment reduces IPV while others suggest it may increase it [18–20]. However, multiple studies indicate that factors such as economic empowerment, education, legal awareness, property ownership, freedom of movement, self-esteem, and belonging to an affluent background can significantly reduce the prevalence of emotional, sexual, and physical IPV [21–24]. A woman’s experience of IPV often reflects her household’s capacity to either foster or hinder her empowerment, as IPV typically indicates a lack of supportive and equitable power dynamics [25]. In contrast, the absence of IPV may suggest a more equitable environment that fosters both women’s empowerment and well-being [26].

As primary caregivers, women’s empowerment can play a crucial role in child health outcomes, as empowered mothers are better able to access resources and provide adequate nutrition, which is essential for child development [27]. Specific dimensions of empowerment, such as decision-making and mobility, have been linked to improved child growth and survival [28]. Additionally, the negative effects of IPV can indirectly impair a woman’s caregiving abilities, further compromising child health [29].

Child development is multidimensional, encompassing communication, motor, problem-solving, and personal-social skills that are highly sensitive to the caregiving environment [29, 30]. IPV can affect these domains through multiple mechanisms: biologically, through stress-related neuroendocrine changes that impair fetal and infant brain development; psychologically, by increasing maternal anxiety and reducing emotional availability; and socially, by limiting support, stimulation, and secure attachment [31].

We conceptualized empowerment as “freedom from family domination,” reflecting women’s autonomy in household decision-making and resistance to coercive family control [16, 17] a salient dimension in Pakistan’s extended-family context. Rather than a mediator, empowerment may serve as a protective moderator that buffers these adverse effects by enabling women to mobilize resources and sustain responsive caregiving despite IPV exposure [27, 32, 33]. This aligns with resilience frameworks suggesting that empowerment can mitigate, rather than explain, the adverse impact of IPV on child well-being [29].

Despite the growing recognition of the effects of IPV on child health, most studies from LMICs have focused on physical growth, with limited research exploring motor and cognitive development [31]. To date, there has been limited research examining the relationship between IPV, women’s empowerment, and child developmental outcomes in Pakistan, with existing studies primarily focusing on child nutrition [9, 14, 34]. Therefore, we aimed to investigate the association between IPV and various domains of child development including communication, motor skills, problem-solving abilities, and social skills. Furthermore, we explored if women’s empowerment (i.e., freedom from family domination) moderated the relationship between IPV and child developmental outcomes.

## Methods

### Study setting

#### Participant recruitment

This study used secondary data originally collected in a randomized controlled trial aimed at assessing the Healthy Mother -- Happy Baby (HMHB), a psychosocial intervention aimed to reduce symptoms of anxiety among 1,200 pregnant women from Holy Family Hospital (HFH), a large public healthcare facility in Rawalpindi, Pakistan [35]. A team of female assessors recruited pregnant women during their initial prenatal visits at the Department of Obstetrics and Gynaecology. This parent study lasted between from April 2019 to October 2022. Data collection of child outcomes was initiated midway through the main study. Therefore, among the 756 participants enrolled in the original HMHB and completed the postpartum assessment, only 400 were included in this sub-study (*n* = 198 receiving usual care and *n* = 202 receiving the trial’s intervention). Baseline characteristics were largely comparable between those included in the sub-study and the rest of the cohort, suggesting minimal risk of selection bias (Supplementary Table 1). By random chance, women enrolled after the substudy began (therefore included in it) were more likely to be empowered (81.8% vs. 80.0% in those enrolled earlier; *p* = 0.01) and to have a higher monthly household income level (55.7% vs. 48.1%; *p* = 0.04). Of the 400 women who completed child development measures during their six-week postpartum visit, we excluded three women who had missingness on income variable. Our final sample size is 397 (99.25% of available sample enrolled in the substudy).

#### Screening and inclusion criteria

The study incorporated a multi-tiered process for inclusion and exclusion during the enrolment phase. In the first tier, potential participants had to meet the following criteria: being at ≤ 22 weeks of gestation, at least 18 years old, residing within a twenty-kilometre radius of HFH, and having a basic understanding of Urdu. Those who met these criteria and expressed willingness to participate were asked to provide informed consent prior to further screening. Individuals were excluded if they reported significant learning disabilities, or severe psychiatric disorders, medical conditions, or maternal complications. The third level of screening involved assessing potential participants for at least mild anxiety using the Hospital Anxiety Depression Scale (HADS) screening tool [36]. Those who scored ≥ 8 on the HADS anxiety sub-scale (indicating at least mild anxiety) underwent interview to rule out clinical depression conducted by a trained assessor using the Structured Clinical Interview for DSM V Diagnoses (SCID) [37]. Women meeting the criteria for a major depressive episode (MDE) at baseline were not included. This study is a secondary analysis of a trial that specifically recruited women with at least mild perinatal anxiety; accordingly, the present analyses examine variation in IPV exposure and infant developmental outcomes within this predefined higher-risk cohort rather than the general population.

### Dependent and independent variables

#### Perinatal intimate partner violence (IPV)

At six weeks postpartum, participants completed a 10-item scale from the Pakistan Demographic and Health Survey assessing exposure to physical (7 items) and emotional (3 items) IPV occurring from the beginning of pregnancy up to the six-week postpartum visit [38]. Answer options for each item were yes (score 1) or no (score 0). The total score ranged from 0 to 10. Higher scores indicated exposure to more types of physical or emotional IPV (see Supplemental Table 2 for the DHS IPV questions). IPV was measured from the beginning of pregnancy to six weeks postpartum to capture overall perinatal exposure, as both prenatal and early postnatal violence may influence maternal wellbeing and caregiving. Sexual IPV was not assessed due to ethical and cultural sensitivities during pregnancy interviews.

#### Child development (ASQ-3)

Child development was measured using the Ages and Stages Questionnaires version 3 (ASQ-3) [39]. The ASQ-3 is a comprehensive checklist of developmental status, standardized for children 1 to 66 months with age-appropriate questionnaires which were translated to Urdu using the World Health Organization seven-step guide for translation and cultural adaptation [40]. For early ages there are questionnaires for every two-month interval of increasing age. We used a two-month questionnaire which can assess development among infants aged between 1 month 0 days and 2 months 30 days. The ASQ-3 has five subscales: communication, gross motor, fine motor, problem- solving and personal-social. Each questionnaire contains 30 items that are age specific (six for each subscale) written in a simple Urdu. For each question, the answer options were ‘yes (score 10)’, ‘sometimes (score 5)’, or ‘not yet’ (score 0). After adding item scores within a domain, the scores in each domain ranged from 0 to 60, with high scores indicating better development. The questionnaires can be completed by caregivers or by a professional in a “home procedure” [39], where a professional plays an active part in the assessment by providing necessary material for the direct assessment of skills during the sessions. We administered the home-procedure version at the hospital.

#### Women’s empowerment

Women’s empowerment was assessed using four items in a subscale corresponding to “freedom from family domination” both at baseline and at six weeks postpartum [17]. We used three items from this four-item scale. They asked whether, in the past year, her husband or other family member(s) had taken (1) her money against her will; (2) her land, jewellery, poultry, or livestock against her will; (3) prevented her from visiting her natal home; or (4) prevented her from working outside the home. Women answering “no” to all four were considered empowered (coded 1), whereas answering “yes” to any of the items was considered non-empowered (coded 0). In the baseline assessment of our study, the fourth item (family preventing her from working outside the home) was accidently omitted, so we considered a woman being empowered if she responded “no” to the first three questions (coded 1).

#### Covariates

For baseline characteristics, women’s age as well as HADS anxiety and depression subscale scores were measured on a continuous scale. Binary variables included women’s education (> or ≤ 8 years), gravidity (primigravida or multigravida), previous pregnancy loss including miscarriage or stillbirth (yes or no), and exposure to any physical violence (perpetrator not specified) in the three months prior to the baseline visit (yes or no). Monthly household income was dichotomized as below or above the poverty line, based on the World Bank’s definition of USD 2.15 per person per day, which corresponded to 18,987 PKR per month in 2019 [41]. For child characteristics, we included sex (male or female), low birthweight (< 2.5 kg vs. normal), and preterm birth (< 37 weeks vs. full-term).

### Statistical analysis

We first calculated mean scores and standard deviations (SDs) for the five ASQ-3 domains, which were different from those obtained in the US general population [42], (Supplementary Table 3). Based on mean scores and SDs in our sample (*n* = 397), we calculated our own cut-off scores of the ASQ-3 domains that define normal, a monitoring zone, and developmental delay. Consistent with the ASQ-3 manual, we used scores equal to or lower than 2 SDs below the mean (≤ mean – 2SD) indicating developmental delay [43]. Scores higher than this cut-off indicate developmental delay (> mean – 2SD) and equal to or lower than 1 SD below the mean (≤ mean – 1SD) indicated borderline developmental delay, whereas the scores higher than 1SD below the mean (> mean – 1SD) indicated normal development. Due to small sample size, we combined borderline and developmental delay to represent developmental delay, and created a dichotomized variable for each domain.

We calculated descriptive statistics for participant characteristics measured at baseline and at delivery, and conducted correlation analyses among the variables of interest (i.e., IPV score, empowerment, and developmental delay). Then, we performed bivariate logistic regression analyses to examine associations between participant characteristics and the five domains of the ASQ-3. Considering both theoretical and statistical associations, exposure to physical violence (not specific to IPV), monthly household income, low birthweight, preterm birth, and treatment (i.e., intervention vs. control arm) were included in further regression models as potential confounders. The intervention did not target IPV or child developmental outcomes directly; however, because participants were drawn from both trial arms, we adjusted for treatment allocation in all regression models to account for any indirect effects of the intervention on maternal psychosocial functioning. Using logistic regression models, we investigated if the number of IPV types a woman was exposed to in the perinatal period was associated with child developmental delay, adjusting for physical violence at baseline, monthly household income, low birthweight, preterm birth, and treatment.

Finally, we created an interaction term between the IPV score and the potential moderating variable, women’s empowerment at six weeks postpartum. Then, for each domain of the ASQ-3, we conducted logistic regression analyses to test effect modification of women’s empowerment in the household on the association between IPV exposure and child development. Wald F-tests were used to identify models with significant moderating effects (*p* < 0.05). The models with significant interaction terms are graphically illustrated to show how associations between IPV and child development varied by levels of women’s empowerment. All analyses were conducted using STATA 19 (Stata Corp, College Station, TX), and *p* < 0.05 was considered statistically significant.

### Ethics

This research underwent a review and received approval from the Institutional Review Boards (IRB) at Johns Hopkins Bloomberg School of Public Health and an National Institute of Mental Health appointed Data Safety and Monitoring Board in the US. In Pakistan, it received approval from the Human Development Research Foundation and Rawalpindi Medical University. Prior to their involvement in this study, participants provided informed written consent.

## Results

Participant mean age was 25.1 (SD = 4.6; Table 1). All the women had at least mild anxiety symptoms with mean HADS scores for anxiety of 11.2 (SD = 1.8). About 60% of women had higher than middle school educational attainment (> 8 years), 30% were pregnant for the first time, and 40% experienced a miscarriage or stillbirth in a prior pregnancy. Approximately 10% reported exposure to any physical violence (irrespective of the perpetrator) in the past three months prior to baseline measurement. A total of 87% of women were classified as empowered at baseline. Slightly more than half of women (56%) had a monthly household income above the poverty line. Relatively equal proportions of women gave birth to male (47%) and female (53%) children. The prevalence of low birthweight and preterm birth was 15% and 23%, respectively.

**Table 1:** Participant baseline characteristics and birth assessment results (*n* = 397)

Participant characteristics	
Baseline	Mean (SD)
Age	25.1 (4.6)
HADS anxiety score	11.2 (1.8)
HADS depression score	6.9 (2.8)
	n (%)
Education: > Middle school (> 8 years) (vs. ≤ middle school)^†^	238 (60.0)
Gravidity: Primigravida (vs. multigravida)	120 (30.2)
Had previous miscarriage or stillbirth (vs. none)	160 (40.3)
Exposure to any physical violence (vs. none)	38 (9.6)
Empowered in household: (vs. not empowered)	345 (86.9)
Monthly household income: Middle (≥ 18,987 PKR) (vs. low)	221 (55.7)
Treatment allocation: Intervention arm (vs. control arm)	199 (50.1)
Birth assessment	
Child sex: Male (vs. female)	187 (47.1)
Low birthweight (< 2.5 kg) (vs. normal)	59 (14.9)
Preterm birth (< 37 weeks) (vs. term)	90 (22.7)
At six-week postpartum	
Empowered in household	323 (81.4)
	Mean (SD)
IPV scores	0.88 (1.76)

Values are presented as mean (SD) for continuous variables and n (%) for categorical variables

^†^ Middle school in Pakistan starts after nine years of schooling so that greater than middle school is equivalent to > 8 years of education

The proportion of empowered women slightly decreased at six-weeks postpartum (81%), compared to a baseline (87%). On average women in our sample (*n* = 397) reported experiencing approximately one type of IPV event (out of 10 types, mean = 0.9, SD = 1.8) since the start of pregnancy, as measured at six weeks postpartum. The proportions of developmental delay in the five domains ranged from 7% to 14%.

IPV exposure and the developmental delays in the five domains were consistently positively associated (Table 2). As expected, there were no significant differences between intervention and control arms in IPV exposure or any ASQ-3 domain scores at six weeks postpartum (all *p* > 0.10). No interaction effects between treatment allocation and IPV or empowerment were detected; therefore, treatment was retained as an adjustment variable rather than a moderator in the final models. After adjusting for exposure to physical violence at baseline, monthly household income, low birthweight, preterm birth, and treatment allocation, exposure to one additional type of IPV was associated with a 1.22–1.30 times higher odds of developmental delay across the five ASQ-3 domains compared with those not exposed to IPV during the perinatal period.

**Table 2:** Association between exposure to intimate partner violence (IPV) and child development measured at six-weeks postpartum

Domains	Developmental delay^†^, *n* (%)	Crude OR (95% CI)	Adjusted OR (95% CI)^‡^
Communication	33 (8.3)	1.18 (1.01, 1.38)^*^	1.22 (1.02, 1.47)^*^
Gross motor	45 (11.3)	1.22 (1.06, 1.39)^**^	1.30 (1.10, 1.53)^**^
Fine motor	29 (7.3)	1.20 (1.03, 1.41)^*^	1.26 (1.04, 1.52)^*^
Problem solving	54 (13.6)	1.17 (1.02, 1.34)^*^	1.22 (1.04, 1.43)^*^
Personal-social	48 (12.1)	1.17 (1.02, 1.35)^*^	1.24 (1.06, 1.46)^**^

^†^ Includes borderline and developmental delay. Scores in the borderline zone are ≤ 1.0 but > 2.0 SD below the mean. Scores higher than the monitoring zone indicate typical development. Scores in the monitoring zone may need further investigation. Scores ≤ 2.0 SD below the mean indicate a possible delay in development (further assessment with a professional is recommended)

^‡^ Adjusted for exposure to physical violence at baseline, monthly household income, treatment allocation (i.e., intervention vs. control arms), low birthweight, and preterm birth

^*^p < 0.05; ^**^p < 0.01

When the interaction terms between IPV during perinatal period and women’s empowerment at six weeks postpartum were included in the logistic regression models, the interaction terms were significant for the gross motor domain (*p* = 0.02, Table 3) after adjustment for covariates.

**Table 3:** Moderating effect of women’s empowerment at six weeks postpartum on the association between intimate partner violence (IPV) scores and child development measured at six-weeks postpartum

Domains	OR for interaction term (95% CI) ^†^	*p*-value^‡^
Communication	0.78 (0.52, 1.18)	0.237
Gross motor	0.60 (0.39, 0.92)	0.020
Fine motor	0.68 (0.43, 1.08)	0.104
Problem solving	0.74 (0.52, 1.06)	0.103
Personal-social	0.86 (0.61, 1.21)	0.391

† Interaction term between IPV and women’s empowerment at six weeks postpartum. Models were adjusted for exposure to physical violence at baseline, monthly household income, treatment, low birthweight, and preterm birth

^‡^ *P*-values for the interaction were obtained from the Wald test

**Fig. 1:**
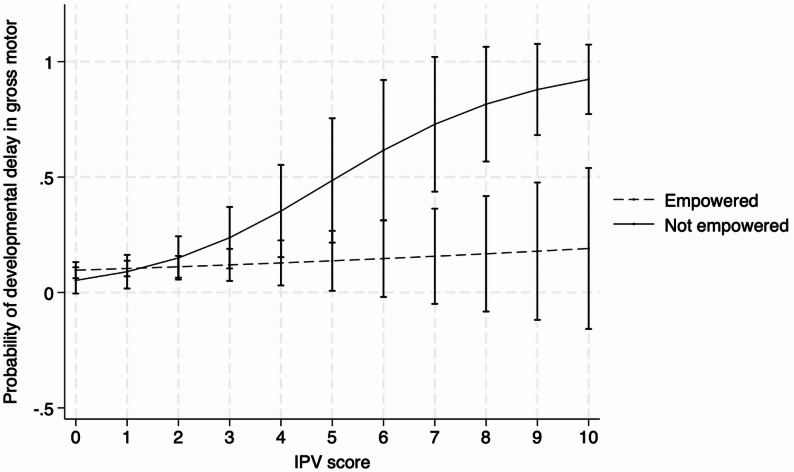
Moderating role of empowerment in the household on the association between exposure to intimate partner violence (IPV) scores and child developmental delay in gross motor measured at six-weeks postpartum. Note: Models were adjusted for exposure to physical violence at baseline, monthly household income, treatment, low birthweight, and preterm birth

Table 4 displays the associations between IPV and child development, stratified by women’s empowerment. We graphically depicted the associations between IPV and developmental delay in gross motor skills, moderated by women’s empowerment (Fig. 1). IPV scores and developmental delay in the gross motor domain was consistently positively related among women who were not empowered in the household, whereas none of the associations were significant among empowered women.

**Table 4:** Association between exposure to intimate partner violence (IPV) and child development measured at six-weeks postpartum, stratified by women’s empowerment at six weeks postpartum

	Among empowered women	Among unempowered women
*n* (%)	323 (81.4)	74 (18.6)
Domains	Adjusted OR (95% CI)	Adjusted OR (95% CI)
Communication	1.09 (0.81, 1.45)	1.26 (0.91, 1.75)
Gross motor	1.11 (0.87, 1.43)	1.62 (1.07, 2.44)^*^
Fine motor	1.12 (0.83. 1.50)	1.44 (0.99, 2.08)
Problem solving	1.12 (0.90, 1.38)	1.42 (1.00, 2.02)
Personal-social	1.19 (0.93, 1.52)	1.16 (0.86, 1.54)

Odds ratios (OR) and 95% confidence intervals (CI) from logistic regression models are shown. Higher ORs indicate greater odds of developmental delay

†Adjusted for exposure to physical violence at baseline, household empowerment measured at baseline, monthly household income, treatment, low birthweight, and preterm birth

^*^p < 0.05

## Discussion

Our findings showed a consistently negative relationship between exposure to IPV and all the facets of child development that we studied, including communication, gross motor skills, fine motor skills, problem-solving abilities, and personal-social skills. These results align with research highlighting how IPV impairs a mother’s ability to care for her child, leading to adverse child developmental outcomes [6, 27, 30, 44]. Importantly, these associations remained significant after adjusting for key covariates, with the adverse effect of IPV on gross motor development observed only among children of women who lacked empowerment. These findings also persisted after accounting for treatment allocation in the parent trial, which had no direct component addressing IPV or child development.

Our findings contribute to the growing body of evidence on the harmful effects of IPV on child development, with our study showing that these effects can be observed in the Pakistani context as early as six weeks of age. A study utilizing data from 15,202 households from 11 LMICs across various regions including Africa, Asia, and Central America reported that exposure to IPV is linked to lower early childhood developmental scores in children aged 3 to 5 years across multiple domains, including learning, literacy, numeracy, socioemotional, and physical milestones [45]. That study focused solely on children aged 3 to 5 years and did not assess outcomes as early as six weeks or include data from Pakistan. Our study expands on these findings by demonstrating that IPV influences all five domains of child development, even in early infancy.

It is also important to consider the potential role of maternal anxiety in interpreting these findings. All participants had at least mild anxiety during pregnancy, which may influence both caregiving behaviors and maternal perceptions of their child’s development. Anxious mothers may provide less responsive or more intrusive care, affecting early bonding and stimulation [29, 30]. Moreover, heightened anxiety could lead to more critical or cautious reporting of infant abilities, potentially inflating associations between IPV and developmental delay. Future studies including mothers with a wider range of anxiety levels would help disentangle these effects.

Results of our analysis of the moderating role of women’s empowerment on the relationship between IPV and child development suggest that children of empowered women were less affected by IPV, whereas children of non-empowered women had significantly worse developmental outcomes when experiencing IPV. While studies specifically examining the interplay between IPV, empowerment, and child development are scarce, there is evidence linking women’s empowerment to improved child anthropometry and nutrition [32]. For instance, research from Pakistan, India, Nepal and Ethiopia and other LIMICs has shown that women’s decision-making power within the household is associated with reduced child stunting, wasting, and underweight, regardless of IPV exposure [27, 33, 46]. Empowered women, who can make decisions and contribute financially, may have greater control over household food procurement and allocation, positively influencing child nutrition and development [14].

,In the Pakistani context, patriarchal norms and gender relations further complicate the dynamics of empowerment and IPV. While increased decision-making power among women has been shown to reduce some forms of IPV, it may also heighten the risk of spousal violence in certain circumstances [27, 47]. Women with fewer resources and lower autonomy are less able to advocate for their children’s well-being, particularly in violence-complicated relationships, compounding the negative effects of IPV on child development [48, 49]. Our study underscores the importance of addressing both IPV and women’s empowerment to improve child developmental outcomes. Moreover, patriarchal social norms that position men as providers of financial support and resources for the family, enforce compliance with gender roles that further exacerbate these challenges [50].

Despite the emphasis in the literature on how maternal experiences of physical and emotional IPV relates to child well-being, there is a notable lack of research examining its impact on a broader set of developmental outcomes, such as cognitive and motor skills. Most studies have primarily focused on outcomes related to food and nutrition. Our study addresses this gap by examining the relationship between IPV, maternal empowerment, and various domains of child development. Our findings highlight that IPV is associated with poorer child development outcomes, and that maternal empowerment may serve as a protective factor against its harmful effects. While depression during pregnancy was an exclusion criterion, we acknowledge that some women may have developed postpartum depression, which could also influence these outcomes.

This study has some limitations. The sample was drawn from a specific population of women of reproductive age experiencing anxiety during pregnancy and delivering in a public hospital in Rawalpindi, Pakistan. This limits the generalizability of the findings to other regions or contexts. Although women with major depressive episodes were excluded, the relatively high mean HADS depression score suggests that many participants may have had mild depressive symptoms, which could independently influence caregiving and child developmental outcomes.It is also possible that maternal anxiety influenced how mothers reported their child’s development; for example, scores in our sample were higher than those reported for the US general population (Supplementary Table 2), which could reflect differences in maternal perceptions or cultural norms around child development. Additionally, we analysed ASQ-3 outcomes as dichotomized variables rather than continuous scores, which may reduce statistical power and precision. This approach, however, was necessary to address non-normality in the continuous scores and is consistent with ASQ-3 guidelines for identifying clinically meaningful developmental delays. Additionally, the conservative nature of the society and the cultural taboos surrounding IPV may have led to its underreporting, which could affect the study’s findings. The IPV measure combined prenatal and postnatal exposure and excluded sexual IPV, which may limit temporal precision and construct completeness. A further limitation is that our household empowerment measure relied on only three indicators, which may not fully capture the complex dimensions of women’s actual agency and decision-making power. Many women in our sample do not work outside the home, lack independent financial assets, and have limited control over property or income, hence answering ‘no’ to questions about taking money or assets may not truly reflect empowerment. This may explain why our study found relatively high rates of ‘empowered’ women compared to what might be expected in this setting. We did not examine differences by child sex, which may be relevant given gender-based disparities in caregiving and development in South Asian contexts.

Empowering women and promoting child nutrition are key components of the 2030 Sustainable Development Goals (SDGs 2 and 5) (United Nations). To progress toward these goals, it is essential to understand the interconnections between women’s empowerment, IPV, and child developmental outcomes. By providing insights into the interplay of these factors in a conservative society like Pakistan, our study offers a clearer understanding of the pathways through which empowerment and IPV can impact child development.

Our findings highlight the urgent need for integrating IPV screening, empowerment counselling, and parenting support into routine maternal health services could help protect both women’s safety and children’s development. This dual approach can protect children from the detrimental consequences of IPV and promote healthier developmental outcomes, contributing to a more equitable and healthier society. As we work toward achieving the broader developmental goals of the SDGs, empowering women and ensuring their children’s well-being should be at the forefront of global and local initiatives.

## Supplementary Information

Supplementary Material 1. Supplementary Table 1. Participant characteristics between those included and excluded in the substudy assessing child developmental outcomes. Supplementary Table 2. Intimate partner violence screening questions, from the Pakistan Demographic and Health Survey. Supplementary Table 3. Mean and standard deviation (SD) of the ASQ-3 scores in study sample in Pakistan and the US children retrieved from the ASQ-3 Technical Report.

## Data Availability

The data used in this study can be accessed at the US National Institutes of Health, National Institute of Mental Health (NIMH) Data Archive: ( https:/nda.nih.gov).
