# Supplementary material for: Associations between exposure to intimate partner violence (IPV) and infant developmental delay: moderating role of women’s empowerment at six weeks postpartum
Source: BMC Public Health. 2026 Feb 9;26:888. doi: 10.1186/s12889-026-26528-9 (PMC12990404; doi:10.1186/s12889-026-26528-9)
Supplement: Supplementary file 1 — Supplementary Material 1. Supplementary Table 1. Participant characteristics between those included and excluded in the substudy assessing child developmental outcomes. Supplementary Table 2. Intimate partner violence screening questions, from the Pakistan Demographic and Health Survey. Supplementary Table 3. Mean and standard deviation (SD) of the ASQ-3 scores in study sample in Pakistan and the US children retrieved from the ASQ-3 Technical Report. [file 12889_2026_26528_MOESM1_ESM.docx]

**SUPPLEMENTARY TABLES**

**Supplementary Table 1**. Participant characteristics between those included and excluded in the substudy assessing child developmental outcomes

| Participant characteristics | Participants enrolled after the substudy began (n=400) | Participants enrolled before the substudy began (n=355) | p-value |
| --- | --- | --- | --- |
| **Baseline** | Mean (SD) | Mean (SD) |  |
| Age | 25.08 (4.57) | 25.55 (4.73) | 0.16 |
| HADS anxiety score | 11.25 (1.80) | 11.03 (2.04) | 0.12 |
| HADS depression score | 6.89 (2.80) | 6.56 (2.69) | 0.10 |
|  | n (%) | n (%) |  |
| Education: > Middle school (> 8 years) (vs. ≤ middle school)^†^ | 239 (59.8) | 192 (54.1) | 0.12 |
| Gravidity: Primigravida (vs. multigravida) | 122 (30.5) | 88 (24.8) | 0.08 |
| Had previous miscarriage or stillbirth (vs. none) | 161 (40.3) | 167 (47.0) | 0.06 |
| Exposure to any physical violence (vs. none) | 38 (9.5) | 44 (12.4) | 0.20 |
| Women’s empowerment (vs. not empowered) | 347 (86.8) | 284 (80.0) | 0.01 |
| Monthly household income: Middle (≥ 18,987 PKR) (vs. low) | 221 (55.7) | 163 (48.1) | 0.04 |
| Treatment allocation: Intervention arm (vs. control arm) | 202 (50.5) | 178 (50.1) | 0.92 |
| **Birth assessment** |  |  |  |
| Child sex: Male (vs. female) | 189 (47.3) | 163 (50.9) | 0.33 |
| Low birthweight (< 2.5kg) (vs. normal) | 61 (15.3) | 38 (11.9) | 0.19 |
| Preterm birth (<37 weeks) (vs. term) | 92 (23.0) | 55 (17.2) | 0.06 |
| **At six-week postpartum** |  |  |  |
| Empowered in household | 326 (81.5) | 291 (82.0) | 0.87 |
|  |  | Mean (SD) |  |
| IPV scores | 0.89 (1.76) | 0.81 (1.77) | 0.57 |

**Supplementary Table 2**. Intimate partner violence screening questions, from the Pakistan Demographic and Health Survey

| **Physical IPV questions:**  1. I am now going to ask you about some situations that are true for many women. Thinking about your husband, since the time that you got pregnant, did your husband… |
| --- |
|  |
| 1a. Push you, shake you, or throw something at you? |
| 1b. Slap you? |
| 1c. Twist your arm or pull your hair? |
| 1d. Punch you with his fist or with something that could hurt you? |
| 1e. Kick you, drag you, or beat you up on purpose? |
| 1f. Try to choke you or burn you on purpose? |
| 1g. Threaten or attack you with a knife, gun, or any other weapon? |
| **Emotional IPV questions:**  2. Now I’m going to ask you about things your husband may have done to you. Since you became pregnant, did your husband… |
| 2a. Say or do something to humiliate you in front of others? |
| 2b. Threaten to hurt or harm you or someone close to you? |
| 2c. Insulted you or made you feel bad about yourself? |

**Supplementary Table 3**. Mean and standard deviation (SD) of the ASQ-3 scores in study sample in Pakistan and the US children retrieved from the ASQ-3 Technical Report

|  | Study sample in Pakistan | | | | US sample | | | |
| --- | --- | --- | --- | --- | --- | --- | --- | --- |
|  | Mean | SD | 1.0SD | 2.0SD | Mean | SD | 1.0SD | 2.0SD |
| Communication | 54.85 | 10.33 | 44.52 | 34.20 | 47.62 | 12.42 | 35.19 | 22.77 |
| Gross motor | 54.24 | 10.52 | 43.72 | 33.20 | 55.32 | 6.74 | 48.58 | 41.84 |
| Fine motor | 54.68 | 10.16 | 44.51 | 34.35 | 29.80 | 9.82 | 39.98 | 30.16 |
| Problem solving | 51.68 | 12.96 | 38.71 | 25.75 | 48.48 | 11.93 | 36.55 | 24.62 |
| Personal-social | 55.63 | 8.81 | 46.82 | 38.01 | 50.57 | 8.43 | 42.14 | 33.71 |

Note: High scores indicate normal child development.
